# Simple intraparenchymal cyst of the cerebellum

**DOI:** 10.11604/pamj.2014.18.337.3619

**Published:** 2014-08-27

**Authors:** Jawad Laaguili, Brahim El Mostarchid

**Affiliations:** 1 Department of Neurosurgery, Mohamed V Military Teaching Hospital, Rabat, Morocco

**Keywords:** Intraparenchymal cyst, cerebellum, MRI

## Image in medicine

We present a magnetic resonance imaging of a 38 old woman, admitted with clinical symptoms of expansive cerebellar lesion. No mural nodule and no enhancement after gadolinium injection were noted. She was operated upon and an anatomico-pathological diagnosis of a simple cyst was made. Cysts of the posterior fossa are common in the literature, but the simple cyst is rare. Considered in the differential diagnosis of these cysts: (hemangioblastoma, astrocytoma cystic, hydatid cyst ...). The postoperative course was uneventful after surgery.

**Figure 1:**
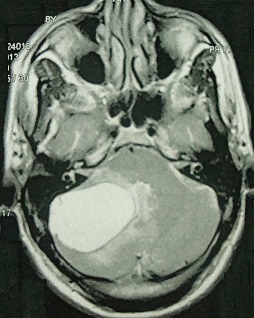
Brain MRI, T2-weighted axial sequence. High intensity signal. Simple cyst compressing the cerebellar vermis and fourth ventricle

